# Crystal structures reveal N-terminal Domain of *Arabidopsis thaliana* ClpD to be highly divergent from that of ClpC1

**DOI:** 10.1038/srep44366

**Published:** 2017-03-13

**Authors:** Chinmayee Mohapatra, Manas Kumar Jagdev, Dileep Vasudevan

**Affiliations:** 1 Institute of Life Sciences, Bhubaneswar - 751023, Odisha State, India

## Abstract

The caseinolytic protease machinery associated chaperone protein ClpC is known to be present in bacteria, plants and other eukaryotes, whereas ClpD is unique to plants. Plant ClpC and ClpD proteins get localized into chloroplast stroma. Herein, we report high resolution crystal structures of the N-terminal domain of *Arabidopsis thaliana* ClpC1 and ClpD. Surprisingly, AtClpD, but not AtClpC1, deviates from the typical N-terminal repeat domain organization of known Clp chaperones and have only seven α-helices, instead of eight. In addition, the loop connecting the two halves of AtClpD NTD is longer and covers the region which in case of AtClpC1 is thought to contribute to adaptor protein interaction. Taken together, the N-terminal domain of AtClpD has a divergent structural organization compared to any known Clp chaperones which hints towards its specific role during plant stress conditions, as opposed to that in the maintenance of chloroplastic homeostasis by AtClpC1. Conservation of residues in the NTD that are responsible for the binding of the cyclic peptide activator - Cyclomarin A, as reported for mycobacterial ClpC1 suggests that the peptide could be used as an activator to both AtClpC1 and AtClpD, which could be useful in their detailed *in vitro* functional characterization.

Plants being sessile organisms are at the risk of getting exposed to a multitude of environmental stress conditions which can cause accumulation and aggregation of misfolded protein species. In this context, the plant caseinolytic protease (Clp) complex plays an important role in degradation of accumulated and misfolded proteins in chloroplasts wherein the chaperones aid in ATP-dependent unfolding of protein substrates to be degraded by the associated protease machinery^1^. Like its counterpart in eubacteria, Clp machinery of higher plants also possesses a barrel-shaped protease core, capped by a homo-hexamer of Clp chaperones belonging to the AAA^+^ superfamily of HSP100 proteins^1^,^2^. In higher plants four different Clp chaperones namely ClpB3, C1, C2 and D are known to get localized into chloroplastic stroma and one each in cytoplasm (ClpB1) and mitochondria (ClpB4) of which ClpC1, C2 and D are known to associate with Clp protease barrel^1^,^3^,^4^,^5^.

The function of chloroplastic Clp chaperones in plants does not restrict to housekeeping duties that are essential for chloroplast development and maintenance as performed by ClpC1^6^ and ClpC2, but also include special duties performed during stress-conditions as performed by ClpB3 and ClpD^4^,^7^. AtClpC1 & AtClpC2 are expressed constitutively, whereas constitutive level of ClpD is relatively low and its expression gets upregulated only during stress conditions such as high salt, dehydration, cold, darkness-induced etiolation and senescence^7^,^8^,^9^,^10^, possibly hinting towards their different substrate preferences. In fact, ClpD was originally named as Early Responsive to Dehydration 1 (ERD1), suggesting its increased expression in response to dehydration. Over-expression of rice ClpD1 protein was found to enhance tolerance to salt and desiccation stresses in transgenic *Arabidopsis* plants^11^.

AtClpC1 and AtClpC2 share 92% identity at the amino acid sequence level, indicating functional overlap. However, the two proteins share only about 48% sequence identity with AtClpD^12^. ClpC1 is highly conserved among the various plant species with more than 90% sequence identity, suggesting high functional importance in plants, whereas, ClpD which has been reported only in plants^13^,^14^ is less conserved across plant species (up to as low as 70% sequence identity). The structured, mature forms of AtClpC1/C2/D consist of three distinct domains such as a short N-terminal domain (NTD) and two ATPase domains^10^ (Fig. 1), a feature very similar to bacterial ClpC proteins^15^. The ATPase domains are essential to bring about ATP-dependent unfolding of proteins to be degraded by the protease core.

Interestingly, more sequence variation between AtClpC1/C2 and AtClpD occurs at the N-terminal domain (34% identity) as compared to the remaining stretch (53% identity). It is the N-terminal domain of Clp chaperones that comes into direct contact with adaptor proteins (if any). The N-terminal domain of ClpC1 is known to perform the regulatory function of substrate recognition, either directly or indirectly through the adaptor proteins ClpS1/ClpF for which it becomes a binding platform^16^,^17^,^18^, whereas no adaptor proteins have so far been reported for ClpD. Even though *in vitro* ATPase activity level of AtClpD has been reported to be lower compared to AtClpC2^14^, it appears that it is the regulatory N-terminal domain and its structure that primarily differentiates the two chaperones, as a primary requirement to recognize and recruit different protein substrates. So we wanted to know whether the N-terminal domain structures of AtClpC1 and AtClpD are considerably different. Here we report and analyse the crystal structures of AtClpC1 residues 94 to 238 and AtClpD residues 79 to 233 (hereafter mentioned as AtClpC1 NTD and AtClpD NTD respectively) that correspond to the ClpC NTD of eubacteria.

## Results and Discussion

### AtClpC1 NTD structure

**Figure 2:**
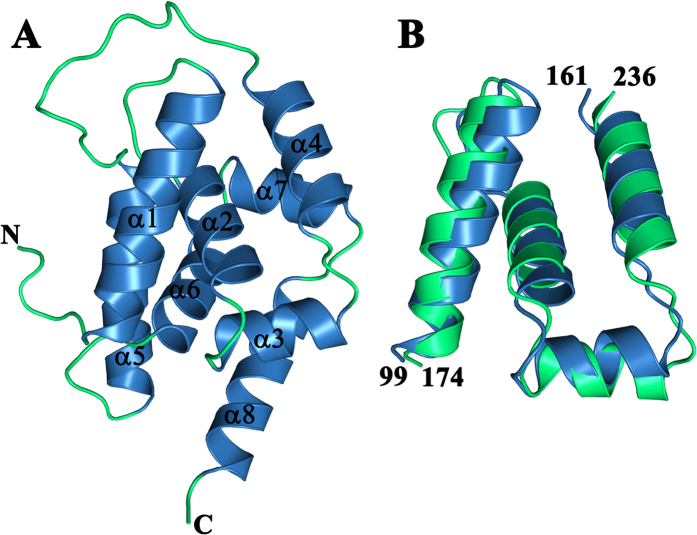
Structure of AtClpC1 N-terminal domain. (**A**) Cartoon representation of the structure of *A. thaliana* ClpC1 NTD with the helices (blue) and loops (green). The N and C termini as well as the eight α-helices are labeled. (**B**) Structural alignment of AtClpC1 residues 99–161 (blue) with residues 174–236 (green).

The 1.20 Å structure showed AtClpC1 N-terminal domain (residues 94–238; Fig. 2A) to be a α-helical domain, having 66% helical content and made up of eight tightly packed α-helices: α1 (E100-L116), α2 (T123-E133), α3 (I137-M145), α4 (L149-I160), α5 (P175-L191), α6 (S198-E208), α7 (V212-L220) and α8 (P224-N238). The two central helices: α2 and α6 which are rich in the hydrophobic residues Leu and Ile contribute significantly to the packing. All residues of the NTD, as well as two residues from the linker of C-terminal His tag could also be seen in the electron density, allowing a complete model to be built. In addition, five phosphate ions were also fit into the density. The final model was refined to an *R* factor of 15.6% and an *R*_free_ of 17.5% for all data between 17.54 Å and 1.20 Å resolution. Ramachandran plot values revealed no outliers for the structure. The data collection and refinement statistics can be found in Table 1.

The fold of AtClpC1 NTD contains two repeats of a 4-helix motif that share 46% sequence identity and 84.1% similarity in a region of 63-residue overlap. A 14-residue long loop between α4 and α5 connects the two repeats. The two halves that stay in close proximity are related by a pseudo two-fold axis of symmetry. Residues 99–161 and 174–236 superpose with a r.m.s. deviation of about 1.77 Å for 46 Cα atoms (Fig. 2B). Strong sequence identity and conservation of secondary structure between the two halves suggest the possibility of an early evolutionary gene duplication event that could have resulted in this repeated motif, as has been predicted for the N-terminal domain of bacterial ClpA and other ClpC proteins^19^. A query to the DALI database^20^ and PDBeFold server^21^ with AtClpC1 NTD structure coordinates yielded NTD of mycobacterial ClpC1 and other Gram-positive bacterial ClpC as the most similar structures. It also showed structural similarity to AtClpT1/T2 proteins, as well as the NTD of malarial and bacterial ClpB proteins and bacterial ClpA proteins.

### AtClpD NTD structure

**Figure 3:**
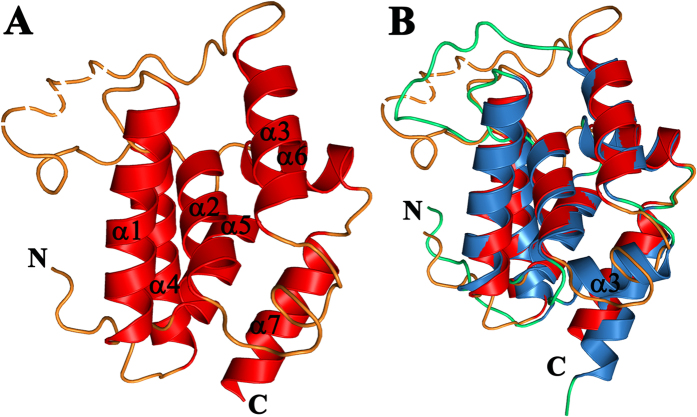
Structure of AtClpD N-terminal domain. (**A**) Cartoon representation of the structure of *A. thaliana* ClpD NTD with the helices (red) and loops (orange). The N and C termini as well as the seven α-helices are labeled. The four-residue long stretch in the long middle loop (that could not be seen in electron density) was fitted into the structure and is shown with intermittant gaps. (**B**) Structural alignment of AtClpD NTD (red helices and orange loops) with AtClpC1 NTD (blue helices and green loops). The N and C termini as well as α3 of AtClpC1 NTD are labeled.

The 1.6 Å structure of AtClpD N-terminal domain (residues 79–233; Fig. 3A) revealed a α-helical structure, having 61% helical content. The domain is made up of seven helices: α1 (E85-L101), α2 (T106-E118), α3 (I132-D144), α4 (I170-M186), α5 (P191-V203), α6 (S207-L215) and α7 (M219-K231). The crystal structure included two molecules in an asymmetric unit. The two molecules had almost identical structures; aligning with an r.m.s. deviation of 0.19 Å for 112 Cα atoms. One of the molecules had better electron density and hence could fit more residues into the structure. For this molecule, all residues of the NTD, except four in the long middle loop and one at C-terminus could be fit into the density. In addition, four residues from the N-terminal linker stretch after GST-tag cleavage could also be fit into the structure. The molecule had an average B value of 21.91 Å^2^. The second molecule did not have any visible electron density for eleven residues of the middle loop and two residues of the C- terminus and had a slightly higher average B value of 26.75 Å^2^. The final model was refined to an *R* factor of 20.5% and an *R*_free_ of 22.3% for all data between 37.87 Å and 1.6 Å resolution. There were no Ramachandran plot value outliers in the structure. The data collection and refinement statistics can be found in Table 1.

**Figure 4:**
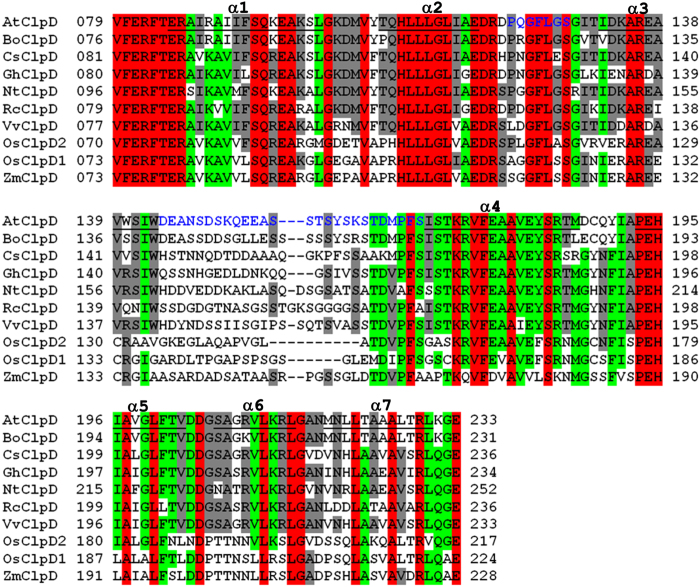
Sequence alignment of AtClpD NTD with the NTD of putative ClpD from different plants. The sequences have been aligned using ClustalW. For AtClpD, the residues forming helices are underlined and the helices are labelled α1 to α7. The residues conserved among all ten sequences are highlighted in red, those conserved among eight or nine sequences are highlighted in green and those conserved in five, six or seven sequences are highlighted in grey. The residues of regions that are substantially different in AtClpD structure as compared to ClpC have been given in blue. Bo, Cs, Gh, Nt, Rc, Vv, Os and Zm corresponds to *Brassica oleracea, Citrus sinensis, Gossypium hirsutum, Nicotiana tabacum, Ricinus communis, Vitis vinifera, Oryza sativa* and *Zea mays* respectively.

**Figure 5:**
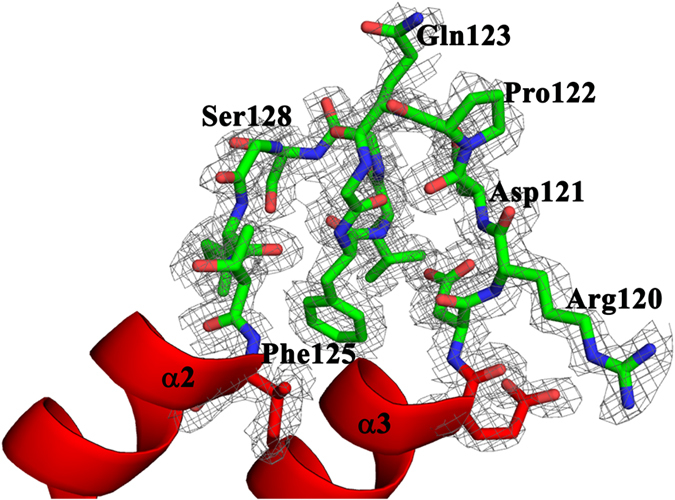
Electron density map for the loop region between helices α2 and α3 in the crystal structure of AtClpD. The Fo-Fc omit map for the residues in the loop region between helices α2 and α3, contoured to 1.0 sigma is represented in grey colour. Helices and loop residues are labeled.

The structure reveals a longer middle loop for AtClpD NTD when compared to AtClpC1 NTD. This feature was obvious when the NTD sequences were aligned to each other. The crystal structure additionally shows the 26-residue long middle loop to take a different conformation compared to the 14-residue long loop of AtClpC1 NTD (Fig. 3B). Interestingly, the sequence of this long middle loop is the least conserved across the ClpD isoforms from different plant species, with its length varying between 19 and 29 residues in the sequences we analyzed (Fig. 4). Also, unlike in AtClpC1, the two halves of AtClpD NTD do not form the typical four-helix motif repeats. In AtClpD, residues 122–128, which makes the short stretch corresponding to α3 of AtClpC1 NTD forms a loop instead of helix (Fig. 3B). The loop region spanning residues Asp119 to Thr131 had unambiguous electron density in the difference Fourier omit map, supporting the absence of a helix (Fig. 5). To rule out the possibility of a deformity caused due to packing in *P*2_1_ space group, we have looked at the packing of molecules within the crystal (Fig. S1). The loop in question of AtClpD structure that corresponds to α3 of ClpA/B/C family proteins is not in close contact with the adjacent molecules in the crystal, thereby ruling out the possibility of a crystallographic artefact. In addition, the structural alignment of AtClpD NTD with AtClpC1 NTD (Fig. 3B) shows the loop to be projecting outwards as compared to the helix of AtClpC1 NTD, which may not have happened if indeed adjacent molecules of AtClpD NTD come in close contact due to crystal packing in this region and deforms the secondary structure. Also, the two molecules of ClpD NTD in the asymmetric unit align with a minimal r.m.s. deviation of about 0.19 Å and the loop in question aligns very well for the two molecules (Fig. S2). There is very little chance that a crystal packing deformity will affect the two molecules the same way when they are not related by symmetry (Fig. S2). The fact that there are helix breaker residues such as a Pro and two Gly within this stretch supports the experimental evidence for the presence of loop instead of a helix. Though only semi-conserved, the corresponding stretch in ClpD isoforms from other plants also does not seem to support the formation of a helix (Fig. 4). The presence of longer middle loop and the absence of a helix in the stretch corresponding to α3 of AtClpC1 NTD make AtClpD NTD very different from any known Clp chaperone NTD structures and perhaps might attribute its functional significance of having to recognize specific substrates during stress conditions, without the involvement of any adaptor proteins. This becomes the first structure of a Clp chaperone wherein the NTD does not have a 4-helix motif repeat organization.

A query to the DALI database and PDBeFold server with AtClpD NTD structure coordinates yielded NTD of Gram-positive bacterial ClpC and mycobacterial ClpC1 as the most similar structures. AtClpD NTD showed some degree of structural similarity to AtClpT1/T2 proteins, as well as the NTD of malarial and bacterial ClpB proteins and bacterial ClpA proteins.

### Structural comparison with ClpC NTD

The N terminal domain of structurally characterized prokaryotic ClpA/B/C proteins all have the four-helical structural repeats conserved. The N terminal domain of bacterial Clp chaperones are involved in substrate binding and interaction with adaptor proteins (e.g., MecA, YpbH, and McsB for ClpC and ClpS for ClpA)^22^,^23^,^24^,^25^. Crystal structures of ClpC N-terminal domain from five different bacteria are available in the protein data bank: *Mycobacterium tuberculosis* (PDB id: 3WDB), *Bacillus subtilis* (PDB id: 2Y1Q), *Corynebacterium glutamicum* (PDB id: 2FH2), *Bacillus lehensis* (PDB id: 4P15) and *Clostridium difficile* (PDB id: 3FES).

**Figure 6:**
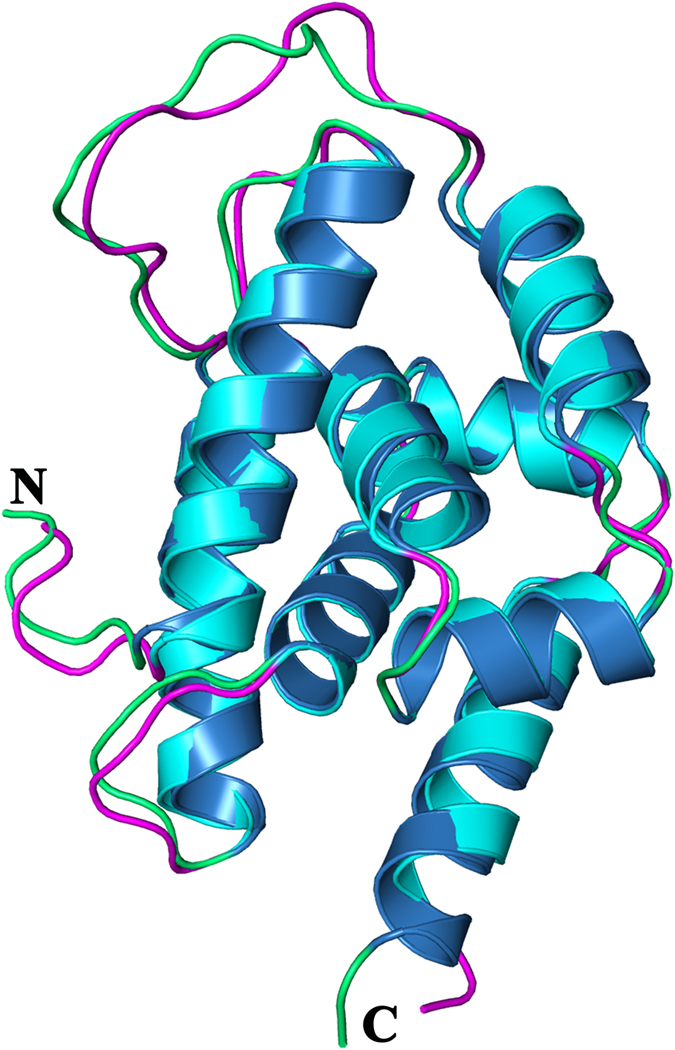
Structural alignment of AtClpC1 NTD with MtClpC1 NTD. AtClpC1 NTD is shown in blue helices and green loops, MtClpC1 NTD is shown in cyan helices and magenta loops. The N and C termini are labeled.

The *A. thaliana* ClpC1 N-terminal domain structure is very similar to the structure of NTD from bacterial ClpC and aligns with r.m.s. deviations of 0.66 Å for 102 Cα atoms with *M. tuberculosis* ClpC1 NTD (PDB id: 3WDB), 0.72 Å for 105 Cα atoms with *B. subtilis* ClpC NTD (PDB id: 2YIQ), 0.76 Å for 103 Cα atoms with *B. lehensis* ClpC NTD (PDB id: 4P15), 1.57 Å for 104 Cα atoms with *C. glutamicum* ClpC NTD (PDB id: 3FH2) and 0.93 Å for 81 Cα atoms with *C. difficile* ClpC NTD (PDB id: 3FES). The structural alignment of AtClpC1 NTD with *M. tuberculosis* ClpC1 NTD has been shown as a representative in Fig. 6.

**Figure 7:**
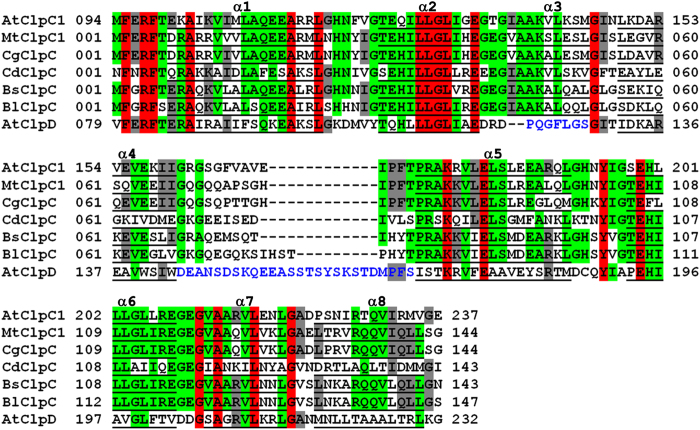
Structure-based sequence alignment of AtClpC1 NTD and AtClpD NTD with bacterial ClpC NTDs. The sequences have been aligned using ClustalW. The residues forming helices are underlined and the helices are labelled α1 to α8 (ClpC numbering). The residues conserved among all seven sequences are highlighted in red, those conserved among five or six sequences are highlighted in green and those conserved in four sequences are highlighted in grey. The residues of regions that are substantially different in AtClpD structure have been given in blue. Mt, Cg, Cd, Bs and Bl corresponds to *Mycobacterium tuberculosis, Corynebacterium glutamicum, Clostridium difficile, Bacillus subtilis* and *Bacillus lehensis*, respectively.

AtClpD NTD structure does not have the four-helical structural repeat organization and it has a longer middle loop. As such, it aligns poorly with the other known ClpC NTD structures which have two halves of four-helix motif. Alignment of AtClpD NTD gives r.m.s. deviations of 0.72 Å for 75 Cα atoms with *C. difficile* ClpC NTD (PDB id: 3FES), 1.05 Å for 99 Cα atoms with *M. tuberculosis* ClpC1 NTD (PDB id: 3WDB), 1.09 Å for 85 Cα atoms with AtClpC1 NTD (PDB id: 3GUI; reported in this work), 1.21 Å for 84 Cα atoms with *B. subtilis* ClpC NTD (PDB id: 2Y1Q), 1.27 Å for 83 Cα atoms with *B. lehensis* ClpC NTD (PDB id: 4P15) and 2.15 Å for 116 Cα atoms with *C. glutamicum* ClpC NTD (PDB id: 3FH2). Figure 7 shows the structure-based sequence alignment of AtClpC1 NTD and AtClpD NTD with bacterial ClpC NTD.

### Structural comparison with ClpA and ClpB NTD

The overall structure of AtClpC1 NTD is similar to that of NTD from bacterial ClpA and ClpB proteins as well. AtClpC1 NTD has its secondary structure organization quite similar to that of *E. coli* ClpA and ClpB -NTD, however the helices and loops of AtClpC1 NTD and those of ClpA/B proteins are oriented slightly different and hence the structure superpose with a relatively poor r.m.s. deviation of 3.82 Å (for 99 Cα atoms) and 7.31 Å (for 81 Cα atoms) to *E. coli* ClpB NTD (PDB id: 1KHY) and *E. coli* ClpA NTD (PDB id: 1R6C), respectively. The NTD of malarial ClpB2 protein (PDB id: 4IOD) also has similar secondary structural features, but superposes with AtClpC1 NTD with a high r.m.s. deviation of 7.14 Å (for 74 Cα atoms). AtClpD aligns with ClpB and ClpA proteins with even higher r.m.s. deviations.

### Structural comparison with ClpT1/T2

**Figure 8:**
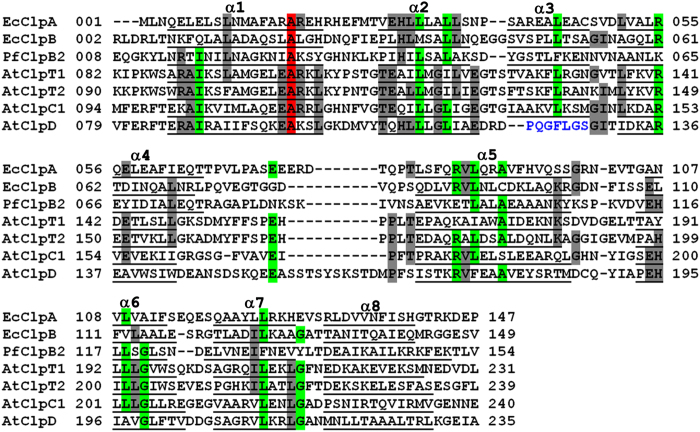
Structure-based sequence alignment of AtClpC1 NTD and AtClpD NTD with ClpA/B NTD and ClpT1/T2 proteins. The sequences have been aligned using ClustalW and the residues forming helices are underlined and helices labelled α1 to α8 (ClpA/B/C/T numbering). The residues conserved among all seven sequences are highlighted in red, those conserved among five or six sequences are highlighted in green and those conserved among four sequences are highlighted in grey. The loop region of AtClpD corresponding to α3 of other structures is given in blue. Ec and Pf correspond to *Escherichia coli* and *Plasmodium falciparum*, respectively.

The chloroplast ClpT1 and ClpT2 proteins show high sequence similarity with each other and are likely derived from ClpC chaperones given their significant sequence similarity (31% sequence identity across 93 to 98 residues) to the NTD of chloroplast ClpC1/C2 chaperones^26^. However, unlike ClpC1, ClpT1 and ClpT2 are unique proteins seen only in higher plants and their interaction with the ClpPR core is predicted to be an adaptation to the plastid proteome and/or Clp protease system of higher plants^26^. Structural characterization has shown ClpT1 and ClpT2 to possess the four-helical structural repeats similar to ClpC NTD^26^. The structure of AtClpT1 (PDB id: 4Y0B) and AtClpT2 (PDB id: 4Y0C) aligns with AtClpC1 NTD with an r.m.s. deviation of 1.03 Å (for 88 Cα atoms) and 1.31 Å (for 84 Cα atoms), respectively and with AtClpD with an r.m.s. deviation of 1.35 Å (for 92 Cα atoms) and 1.44 Å (for 90 Cα atoms), respectively. Structure-based sequence alignment of the NTD of AtClpC1 and AtClpD with AtClpT1, AtClpT2 as well the NTD of *E. coli* ClpA, *E. coli* ClpB and malarial ClpB2 is presented in Fig. 8.

### AtClpC1/D NTD and interaction partners

The NTD of *E. coli* ClpA protein is only about 23% identical to AtClpC1 NTD in its sequence; however, the two proteins seem to have a very similar structural organization. *E. coli* ClpA NTD residues involved in interaction with ClpS are conserved in AtClpC1 NTD as well^16^. *E. coli* ClpS protein is only about 27% identical to AtClpS1 protein with respect to its sequence. The interaction between AtClpC1 and AtClpS1 is thought to be quite similar to that of *E. coli* ClpA and ClpS proteins^16^. After having the AtClpC1 NTD structure, we also presume the mode of interaction between AtClpC1 NTD and AtClpS1 to be similar to that between *E. coli* ClpA and *E. coli* ClpS. The discovery of AtClpF protein as a third partner of the complex^18^ has added another level of complexity and with the current level of understanding, predictions looking at AtClpC1 and AtClpS1 in isolation may not be prudent. In fact, our attempts to obtain a complex of AtClpS1 with AtClpC1 NTD by pull down assays and co-purification of AtClpS1 with monomeric as well as hexameric forms of full-length AtClpC1 were unsuccessful, suggesting the need to have even AtClpF in the complex (Supplemental data). Future studies should focus on the structural characterization of a complex made up of the interacting domains of AtClpC1, AtClpS1 and AtClpF. This would shed light on the exact mode of substrate recognition by the plant Clp machinery.

Unlike AtClpC1 and AtClpC2, AtClpD is not known to have an adaptor protein. AtClpS1 and AtClpF do not interact with AtClpD^16^,^18^. AtClpD not interacting with AtClpS1 has primarily been attributed to the absence of residues present in AtClpC1 that support its interaction with AtClpS1. When the structure of AtClpD NTD is aligned with AtClpC1 NTD, we realize that the long middle loop comes directly over the region which may otherwise contribute to ClpS1 interaction. Therefore, AtClpD not binding to AtClpS1 or AtClpF could at least partly be due to the presence of the long middle loop in AtClpD NTD which would sterically prevent any such interaction. In the functional and hexameric context of AtClpD, the absence of a helix (α3) in the face opposing the longer loop region might also attribute to the inability to interact with the adaptor proteins AtClpF/S1. It is not clear whether the absence of an adaptor protein is compensated by these structural variations.

**Figure 9:**
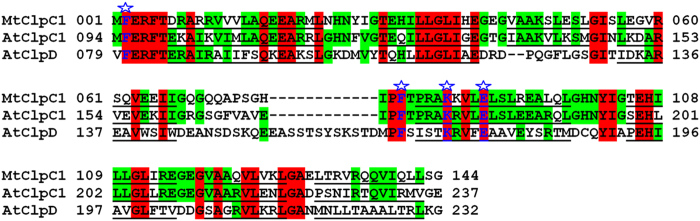
Structure-based sequence alignment of AtClpC1 NTD and AtClpD NTD with MtClpC1 NTD, highlighting the residues involved in Cyclomarin A interaction. The sequences have been aligned using ClustalW and the residues forming helices are underlined. The residues of MtClpC1 NTD involved in Cyclomarin A interaction are shown in blue and with a star on top. The corresponding residues of AtClpC1 NTD and AtClpD NTD are shown in blue. The residues conserved among all three sequences are highlighted in red and those conserved among two sequences are highlighted in green.

**Figure 10:**
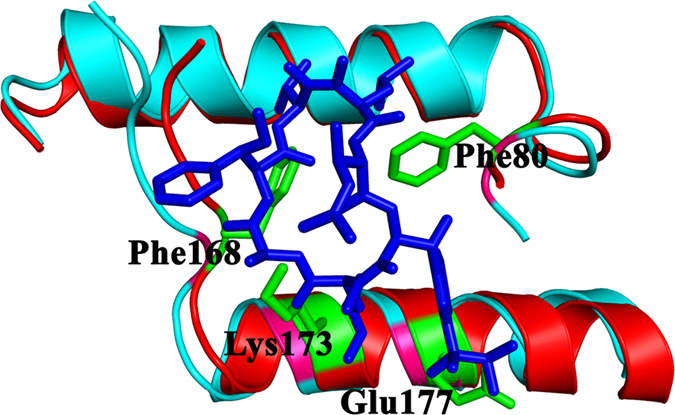
Structural alignment of AtClpD NTD with MtClpC1 NTD in complex with Cyclomarin A. MtClpC1 NTD is shown in cyan and residues involved in Cyclomarin A (CymA) interaction are shown in magenta (coordinates from PDB id: 3WDC). CymA is shown in stick model and in blue. AtClpD NTD is shown in red and the residues which could interact with CymA (Phe80, Phe168, Lys173 and Glu177) are shown as sticks in green and labelled. The region of NTDs coming in the proximity of CymA alone is shown for figure clarity.

While looking for sequence similarity between AtClpC1/C2 NTD and *Mycobacterium tuberculosis* ClpC1 NTD, we found the conserved nature of residues in AtClpC1/C2 that are involved in the interaction of MtClpC1 NTD with the cyclic peptide Cyclomarin A (CymA). Residues Phe2, Phe80, Lys85 and Glu89 of MtClpC1 NTD are responsible for CymA interaction as has been reported in the crystal structure of the complex^27^. A comparison of the crystal structures of MtClpC1-CymA complex and AtClpC1 shows the CymA-interacting residues to be conserved. Phe95, Phe173, Lys178 and Glu182 of AtClpC1 occupy the same position as the residues involved in MtClpC1 NTD interaction with CymA (Fig. 9). So we presume CymA would bind readily to AtClpC1 NTD as well. CymA has been studied functionally and structurally as an anti-TB compound targeting mycobacterial ClpC1 and not active against other Gram-positive bacteria where not all these residues are conserved in the ClpC protein^27^,^28^. It has been proposed that the lack of activity of CymA on Gram-positive bacteria could be due to weaker CymA binding as a result of sequence variation, as well as the presence of the adaptor protein MecA that binds close to the CymA-binding site which is absent in the case of mycobacteria^27^. In the context of plant Clp machinery, though this compound would be of no value as a drug, it could serve as a reagent useful in functionally characterizing the chaperone; more as an activator to the machinery. Interestingly, AtClpD NTD also has all the four CymA-interacting residues conserved with MtClpC1: AtClpD residues Phe80, Phe168, Lys173 and Glu177 (Figs 9 and 10). Due to non-availability of CymA from commercial sources, we could not perform binding or co-crystallization experiments of CymA with AtClpC1/D proteins. MtClpC1 has more recently become an attractive target for anti-TB drug research with peptides of actinomycete origin such as Cyclomarin A, Lassomycin and Ecumicin reported to target the protein’s NTD^28^,^29^,^30^. However, the residues predicted to be involved in MtClpC1 interaction with Lassomycin and Ecumicin based on genome-wide mutational studies with resistant mycobacterial strains^29^,^30^ are not conserved in AtClpC1/C2 as well as AtClpD.

In summary, we have structurally characterized the NTD of AtClpC1 and AtClpD. The structures have also been compared with other known Clp chaperone NTD structures. AtClpC1 resembles all other ClpC chaperone protein NTDs whereas, AtClpD turned out to be a divergent one lacking the typical 4-helix repeat motif that is present in all known Clp chaperone structures. The longer middle loop in its NTD also makes AtClpD different from other known Clp chaperone families. The structural difference in the N-terminus could perhaps be important for AtClpD in specifically recognizing misfolded substrates produced during stress conditions and for subsequent unfolding and proteolysis. An understanding about the structural variations in the NTD of AtClpC1 and AtClpD from this work paves way to further studies involving their interaction with adaptor proteins and/or substrates within the plant chloroplast. We propose Cyclomarin A to be a suitable reagent that would activate plant ClpC/D family proteins and thereby aiding their *in vitro* functional characterization.

## Methods

### Construction of *Escherichia coli* expression plasmids and protein purification

Genes coding for AtClpC1 and AtClpD, optimized for overexpression in *E. coli* were obtained from Genscript (NJ, USA) in pUC57 vector. The DNA sequence coding for the N-terminal domain of AtClpC1 spanning residues 94–238 was amplified by PCR and cloned in frame into a pET22b (Novagen) vector between NdeI and XhoI restriction sites for expression with a non-cleavable carboxyl-terminal hexahistidine tag. The DNA sequence coding for AtClpD NTD spanning residues 79–233 was amplified by PCR and cloned into a pGEX-6P-1 (GE Healthcare) vector between BamHI and XhoI restriction sites for expression with a cleavable N-terminal GST tag. The recombinant proteins were expressed in *E. coli* (BL21/DE3 strain). The expression of AtClpC1 NTD was induced at OD_600_ of 0.4 with 0.5 mM isopropyl β-D-thiogalactopyranoside (IPTG) at 37 °C in 2xYT medium and allowed to proceed for 4 hours. The cell lysate for AtClpC1 NTD was clarified by centrifugation and the supernatant was passed through a HisTrap FF nickel affinity column (GE Healthcare), followed by a HiLoad 16/600 Superdex 75 prep grade column (GE Healthcare). The expression of recombinant AtClpD NTD was induced at OD_600_ of 0.6 with 0.2 mM IPTG at 16 °C in 2xYT medium and allowed to proceed overnight. The cell lysate for AtClpD NTD was clarified by centrifugation, the supernatant passed through a GSTrap FF column (GE Healthcare) and the bound protein eluted from the column for cleavage with PreScission protease (GE Healthcare). In order to separate AtClpD NTD from any excess of the uncleaved GST fusion protein and cleaved off GST, the protein was again passed through GSTrap FF column. The protein was then passed through a HiLoad 16/600 Superdex 75 prep grade column. The eluant from the column was in a buffer containing 20 mM Tris pH 7.5, 150 mM NaCl, 1 mM DTT, and 1 mM PMSF.

### Crystallization and data collection

Crystals of AtClpC1 NTD with carboxyl-terminal hexahistidine tag appeared in sitting drop vapor diffusion plate in 3–4 days in a condition having 1 M ammonium phosphate and 100 mM sodium citrate, pH 5.6. The crystals were transferred into a cryoprotectant solution containing the reservoir solution supplemented with 25% glycerol and then flash cooled in liquid nitrogen. Preliminary diffraction data collection was carried out at the x-ray diffraction facility of Indian Institute of Chemical Technology (IICT), Hyderabad, India on an R-axis IV++ machine equipped with an image plate detector. High resolution dataset was later collected using the Indian synchrotron radiation source at the RRCAT beamline BL21 and recorded with a MarCCD detector. A total of 120 frames of 1° oscillation were collected at a wavelength of 0.9794 Å. The diffraction data were processed using iMOSFLM^31^ and AIMLESS^32^ from the CCP4 suite of programs^33^. The crystal belonged to the orthorhombic space group *P*2_1_2_1_2_1_ with unit cell dimensions a = 40.10 Å, b = 44.29 Å, c = 97.56 Å, and α = β = γ = 90°. One molecule was found per asymmetric unit with a solvent content of ~54%.

AtClpD NTD with carboxyl-terminal hexahistidine tag was highly soluble and did not yield any crystals even after extensive screening. However, crystals of AtClpD NTD (obtained from GST-tagged construct) appeared in sitting drop vapor diffusion plate in 1–2 days in a condition having 20% v/v 2-propanol, 20% w/v PEG MME 2000 and 100 mM MES monohydrate, pH 6.0. The drop containing crystals was overlaid with mineral oil to avoid excessive evaporation of 2-propanol and a cryoprotectant solution containing the reservoir solution supplemented with 20% ethylene glycol was added to the drop and a single crystal flash cooled in liquid nitrogen. High resolution dataset was collected at beamline BM14 of ESRF and recorded with a MarCCD detector. The diffraction data of AtClpD NTD crystal were also processed using iMOSFLM and AIMLESS from the CCP4 suite of programs. The crystal belonged to the monoclinic space group *P*2_1_ with unit cell dimensions a = 38.08 Å, b = 37.31 Å, c = 99.98 Å, α = γ = 90°, and β = 96.08°. Two molecules were found per asymmetric unit with a solvent content of ~39%.

### Structure determination

The crystal structure of AtClpC1 was solved by molecular replacement method using the program Molrep^34^ from CCP4 suite of programs, using the coordinates of *M. tuberculosis* ClpC1 NTD crystal structure in complex with Cyclomarin A (PDB id: 3WDC)^27^, but using only the NTD coordinates as a search model. Model building and structure refinement were carried out using COOT^35^ and Refmac5^36^ from CCP4 suite of programs. The crystal structure of AtClpD NTD was solved by molecular replacement method with Molrep program, using the coordinates of AtClpC1 NTD crystal structure (PDB id: 5GUI) as a search model. Model building and structure refinement of AtClpD NTD were carried out similar to AtClpC1 NTD. The structures were analyzed for stereochemical quality with the help of Ramachandran Plot from the program PROCHECK^37^. The structure figures were prepared and structural superpositions were prepared and calculated using PyMOL Molecular Graphics System (Schrodinger, LLC).

### Accession numbers

Atomic coordinates and structure factors have been deposited with the Protein Data Bank under the accession codes 5GUI (AtClpC1 NTD) and 5GKM (AtClpD NTD).

## Additional Information

**How to cite this article**: Mohapatra, C. *et al*. Crystal structures reveal N-terminal Domain of *Arabidopsis thaliana* ClpD to be highly divergent from that of ClpC1. *Sci. Rep.* **7**, 44366; doi: 10.1038/srep44366 (2017).

**Publisher's note:** Springer Nature remains neutral with regard to jurisdictional claims in published maps and institutional affiliations.

## Supplementary Material

Supplemental Information

## Figures and Tables

**Figure 1:**
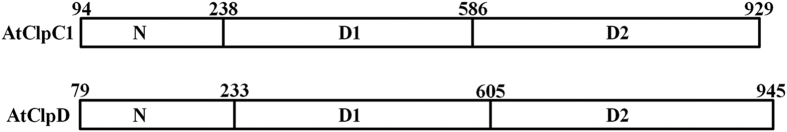
Domain organization of AtClpC1 and AtClpD. The organization of functional domains of AtClpC1 and AtClpD are shown with their respective amino acid residue boundaries. N corresponds to the N-terminal domain, D1 the first ATPase domain and D2 the second ATPase domain.

**Table 1:** Data collection and refinement statistics for AtClpC1 NTD and AtClpD NTD.

Parameter	AtClpC1 NTD	AtClpD NTD
**Data Collection**		
Beamline	RRCAT-BL21	ESRF-BM14
Detector type	Marmosaic 225 mm CCD	Marmosaic 225 mm CCD
Wavelength (Å)	0.9794	0.9537
Data collection temperature (K)	103	100
Space group	*P*2_1_2_1_2_1_	*P*2_1_
a, b, c (Å)	40.10, 44.29, 97.56	38.08, 37.31, 99.98
α, β, γ (°)	90, 90, 90	90, 96.08, 90
Resolution (Å)	17.54–1.20 (1.26–1.20)	37.87–1.60 (1.63–1.60)
*R*_meas_ (%)	7.5 (63.4)	8.8 (78.1)
*I*/σ*I*	9.4 (2.7)	9.8 (2.2)
*CC(1/2*) (%)	99.8 (80.5)	99.5 (71.7)
Total number of reflections	262145	351287
Mosaicity (°)	0.43	0.41
Completeness (%)	98.4 (100)	100 (100)
Multiplicity	4.8 (4.7)	4.2 (4.1)
Wilson B-factor (Å^2^)	11.9	18.2
Matthews coefficient (Å^3^/Da)	2.66	2.00
Solvent content (%)	53.75	38.63
No. of molecules in ASU	1	2
**Refinement**		
No. of unique reflections	54126	37033
R_work_/R_free_ (%)	15.6/17.5	20.5/22.3
Total no. of non-H atoms	1459	2450
No. of water molecules	130	104
No. of ligands	5 phosphate ions	0
Mean B-factor (Å^2^)	18.0	23.0
**R.m.s. deviations**		
Bond lengths (Å)	0.016	0.012
Bond angles (°)	1.931	1.370
**Ramachandran plot values (%)**		
Favoured/Outliers	100/0	100/0

Numbers in parentheses correspond to the last resolution shell. R_meas_ = Σ_h_(n/n − 1)^1/2^ Σ_i_ |I_*i*_(*h*) − <I(*h*)>|/Σ_h_Σ_i_ I_i_(*h*), where I_i_(*h*) and <I(*h*)> are the ith and mean measurement of the intensity of reflection *h*. R_work_ = Σ_h_||*F*_obs_ (*h*)| − |*F*_calc_ (*h*)||/Σ_h_|*F*_obs_ (*h*)|, where *F*_obs_ (*h*) and *F*_calc_ (*h*) are the observed and calculated structure factors, respectively. R_free_ is the R value obtained for a test set of reflections consisting of a randomly selected 5% subset of the data set excluded from refinement.
