# Supplementary material for: Crystal structures reveal N-terminal Domain of Arabidopsis thaliana ClpD to be highly divergent from that of ClpC1
Source: Sci Rep. 2017 Mar 13;7:44366. doi: 10.1038/srep44366 (PMC5347014; doi:10.1038/srep44366)
Supplement: Supplemental Information [file srep44366-s1.doc]

**SUPPLEMENTAL INFORMATION**

**Crystal structures reveal N-terminal Domain of *Arabidopsis thaliana* ClpD to be highly divergent from that of ClpC1**

Chinmayee Mohapatra*, Manas Kumar Jagdev*, Dileep Vasudevan

**Methods**

**Construction of additional *Escherichia coli* expression plasmids and protein purification.** Genes coding for AtClpC1, AtClpD and AtClpS1 optimized for overexpression in *E. coli* and obtained from Genscript in pUC57 vector was used for preparing the additional constructs that were used for pull-down assays and co-purification studies. The DNA sequence coding for mature and structured forms of AtClpC1 (residues 94-929), AtClpD (residues 79-945) and AtClpS1 (residues 45-159), as well as AtClpC1 NTD (residues 94-238) and AtClpD NTD (residues 79-233) were amplified by PCR and cloned in frame individually into pET22b (Novagen) vector, between NdeI and XhoI restriction sites for expression with non-cleavable carboxyl-terminal hexahistidine tag. In addition, the DNA sequence coding for AtClpC1 NTD (residues 94-238) and AtClpD NTD (residues 79-233) were amplified by PCR and cloned into a pGEX-6P-1 (GE Healthcare) vector between BamHI and XhoI restriction sites for expression with a cleavable N-terminal GST tag. All the recombinant proteins were expressed using *E. coli* BL21 (DE3) strain [or else BL21 Star (DE3) strain] in 2xYT medium. The optimized expression conditions for the various constructs used in this work have been summarized in Table S1. The cell lysates for individual proteins expressed with hexahistidine tag were clarified by centrifugation and the supernatants passed through HisTrap FF nickel affinity column (GE Healthcare), followed by a HiLoad 16/600 Superdex 75 prep grade column (GE Healthcare) for AtClpC1/D NTD and AtClpS1, or else a HiLoad 26/600 Superdex 200 prep grade column (GE Healthcare) for AtClpC1/D full-length. The cell lysate for proteins expressed with GST tag was clarified by centrifugation, the supernatants passed through GSTrap FF column (GE Healthcare) and the bound proteins eluted from the column. All purifications were carried out with the help of AKTA Pure 25 M chromatography system (GE Healthcare) housed in a cold chamber. For GST pull-down assays, the GST tag of the fusion proteins was retained and in other cases, the GST tag was cleaved off with PreScission protease (GE Healthcare). In order to separate AtClpD NTD from any excess of the uncleaved GST fusion protein and cleaved off GST, the protein was again passed through GSTrap FF column. The protein was then passed through a HiLoad 16/600 Superdex 75 prep grade column. The eluant from Superdex gel filtration columns for all the proteins were in a buffer containing 20 mM Tris pH 7.5, 300 mM NaCl, 1 mM DTT and 1 mM PMSF.

| **Sl. No.** | **Construct type** | **Competent cell type** | **OD_600_ at induction** | **IPTG concentration for induction** | **Incubation temperature post induction** | **Incubation duration post induction** |
| --- | --- | --- | --- | --- | --- | --- |
| 1 | AtClpC1 FL (His) | BL21 Star (DE3) | 0.7 | 0.5 mM | 18 ^o^C | 16 hours |
| 2 | AtClpD FL (His) | BL21 Star (DE3) | 0.7 | 0.5 mM | 18 ^o^C | 16 hours |
| 3 | AtClpS1 FL (His) | BL21 (DE3) | 0.6 | 0.5 mM | 37 ^o^C | 4 hours |
| 4 | AtClpC1 NTD (His) | BL21 (DE3) | 0.4 | 0.5 mM | 37 ^o^C | 4 hours |
| 5 | AtClpD NTD (His) | BL21 (DE3) | 0.6 | 0.2 mM | 18 ^o^C | 16 hours |
| 6 | AtClpC1 NTD (GST) | BL21 (DE3) | 0.5 | 0.5 mM | 37 ^o^C | 4 hours |
| 7 | AtClpD NTD (GST) | BL21 (DE3) | 0.6 | 0.2 mM | 16 ^o^C | 16 hours |

**Table S1: Optimized expression conditions for the various constructs.** The over-expression conditions optimized for the various full-length and N-terminal domain constructs used in this work are summarized in the table. Full-length constructs are given as FL and N-terminal domain constructs as NTD. Constructs expressed with a C-terminal, non-cleavable hexahistidine tag or a cleavable, N-terminal GST tag are named with His and GST extensions, respectively within parentheses.

***In vitro* interaction by pull-down assays.** The recombinant proteins used for pull-down assays were all purified by gel filtration chromatography using HiLoad 16/600 Superdex 75 prep grade column as described earlier. GST-AtClpD NTD or GST-AtClpC1 NTD or GST was mixed with AtClpS1 protein having a C-terminal hexahistidine tag, incubated for 90 min at room temperature in a buffer containing 25 mM Tris pH 7.5, 150 mM NaCl, 1 mM DTT, 1 mM EDTA, 10% glycerol, 1 mM PMSF and 0.5% Triton X-100. The individual mixtures were then combined with pre-equilibrated glutathione sepharose 4B resin beads (GE Healthcare) for 60 min at 4 ^o^C. The resin beads with bound proteins were washed thrice and the proteins bound to the beads were eluted by directly boiling with SDS-PAGE gel loading buffer. The individual proteins before pull-down as well as eluants were analyzed by SDS-PAGE, stained with Coomassie Brilliant Blue R 250. In order to rule out interference (if any) due to the bulky GST-tag of the fusion proteins, pull-down assays were also carried out with AtClpD NTD and AtClpC1 NTD wherein the GST tag was cleaved. The NTD proteins without any tag were mixed individually with AtClpS1 protein having C-terminal hexahistidine tag and incubated for 90 min at room temperature in a buffer containing 25 mM Tris pH 7.5, 150 mM NaCl, 1 mM DTT, 10% glycerol, 1 mM PMSF, 20 mM imidazole and 0.5% Triton X-100. The individual mixtures, as well as the NTD proteins by themselves were then combined with pre-equilibrated Talon metal affinity resin beads (Clontech) for 30 min at 4 ^o^C. The resin beads with bound proteins after washing thrice were analyzed as done for GST pull-down assay.

***In vitro* interaction by analytical gel filtration.**  AtClpS1 was mixed with full-length AtClpC1 or AtClpD and incubated for 90 min at room temperature, in a buffer containing 20 mM Tris pH 8.0, 300 mM NaCl, 1 mM PMSF and 1 mM DTT, supplemented with 1 mM EDTA, in order to get predominantly monomeric form of AtClpC1/D. The protein mixtures were subjected to gel filtration chromatography with a Superdex 200 10/300 GL column (GE Healthcare), using a buffer containing 20 mM Tris pH 8.0, 300 mM NaCl, 1 mM PMSF, 1 mM DTT and 0.5 mM EDTA. The same experiment was carried out by mixing AtClpS1 with full-length AtClpC1 or AtClpD and incubating for 90 min at room temperature, in a buffer containing 20 mM Tris pH 8.0, 300 mM NaCl, 1 mM PMSF and 1 mM DTT, supplemented with 2 mM AMP-PNP and 2 mM MgCl_2_, in order to get hexameric form of AtClpC1/D. The protein mixtures were subjected to gel filtration chromatography with a Superose 6 10/300 GL column (GE Healthcare), but with a buffer without EDTA and containing 20 mM Tris pH 8.0, 300 mM NaCl, 1 mM PMSF, 1 mM DTT, 1 mM ATP and 1 mM MgCl_2_. The fractions were analyzed by SDS-PAGE, stained with Coomassie Brilliant Blue R 250.

**Results and discussion**

**Expression and purification of full-length AtClpC1 and AtClpD.** According to previous literature^14^, attempt to express recombinant AtClpC1 in bacteria were unsuccessful and further *in vitro* studies^16,18^ have made use of its homolog AtClpC2, to draw comparisons with AtClpD. Also, recombinant full-length AtClpD has been reported to get expressed along with a truncation version; seen as two nearby bands on SDS-PAGE^14^. However, we were able to express recombinant AtClpC1 and AtClpD in soluble form in bacteria and we also managed to purify those to homogeneity. Perhaps, the usage of codon-optimized genes for bacterial expression helped us in getting the protein expressed and without any truncation. Also, we had avoided the short, unstructured stretch prior to NTD for both the proteins. The proteins however, were not very stable and started degrading fast, even in the presence of protease inhibitor.

**AtClpD becomes a hexamer more readily than AtClpC1.** AtClpC1 and AtClpD were expressed and purified under identical conditions without any EDTA. During purification by gel filtration using HiLoad 16/600 Superdex 200 prep grade column, it was observed that AtClpC1 came mostly as a monomer, with very little dimeric and hexameric forms. However, AtClpD was predominantly coming as a hexamer, along with considerable percentage as monomer and dimer. So at least under recombinant expression and purification conditions, AtClpD seems to become a hexamer more readily than AtClpC1 (Fig. S3 and Fig. S6). Recombinant AtClpD readily forms a hexamer may be because it does not need any additional protein factors such as the adaptor proteins for the stability of its hexameric form; whereas, in the case of AtClpC1, the stability of its hexamer could partly be contributed by the partnering adaptor proteins such as AtClpS1 and AtClpF^18^. The oligomeric forms of both the proteins could be avoided by addition of EDTA in the purification buffer, suggesting it to be an ATP-dependent oligomerization process (Fig. S5A and Fig. S5B).

**AtClpS1 does not make a stable interaction with AtClpC1 NTD.** The first report of AtClpS1^16^ showed it getting pulled down by GST-AtClpC1 NTD, but only in negligible amounts. Our attempt to pull-down AtClpS1-His by GST-AtClpC1 NTD did not show any such interaction (Fig. S4A) and we thought it could partly be due to the interference by bulky GST-tag. To rule out such a possibility, we decided to check whether AtClpS1 would bind more readily if the GST tag of AtClpC1 NTD is removed. Towards this end, we used AtClpS1-His and AtClpC1 NTD (with its GST-tag removed), which was then passed through Talon affinity beads. We could not see any interaction here as well (Fig. S4B). This is not completely unexpected, as more recent literature^18^ suggests the involvement of AtClpF, which we could not get expressed. The NTD of AtClpC1 might need both AtClpS1 and AtClpF as adaptors for a functional complex formation^18^. In our pull-down assays, we did not see any interaction for AtClpD NTD with AtClpS1 (Fig. S4A and Fig. S4B) and that was totally expected as no adaptor proteins have so far been reported for AtClpD.

**AtClpS1 does not co-purify even with full-length AtClpC1 or its hexamer.** Analytical gel filtration chromatography experiments for interaction with AtClpS1 were carried out for full-length AtClpC1 and AtClpD. Interestingly, AtClpS1 did not get co-purified with full-length AtClpC1 monomer, as well as hexamer (Fig. S5A and Fig. S6A). If AtClpS1 was binding to AtClpC1, the early fractions of AtClpC1 should have brought along some amount of AtClpS1. However, the early fractions of AtClpC1 did not show presence of AtClpS1, even in trace amounts. A little of AtClpS1 coming along with the trailing fractions of AtClpC1 is thought to be because of a certain degree of peak overlap due to slow fractionation of AtClpC1. AtClpD also showed no AtClpS1 association; both in monomeric and hexameric form (Fig. S5B and Fig. S6B). It appears that AtClpS1does not interact with AtClpC1 under the *in vitro* conditions we tried. Presence of AtClpF protein seems essential for the formation of a stable complex. It is also possible that, the association with adaptor proteins might require the presence of a substrate. However, the current study does not address these aspects. Further work needs to be carried out to completely understand the interaction between AtClpC1 and AtClpS1.

**Figures**

**
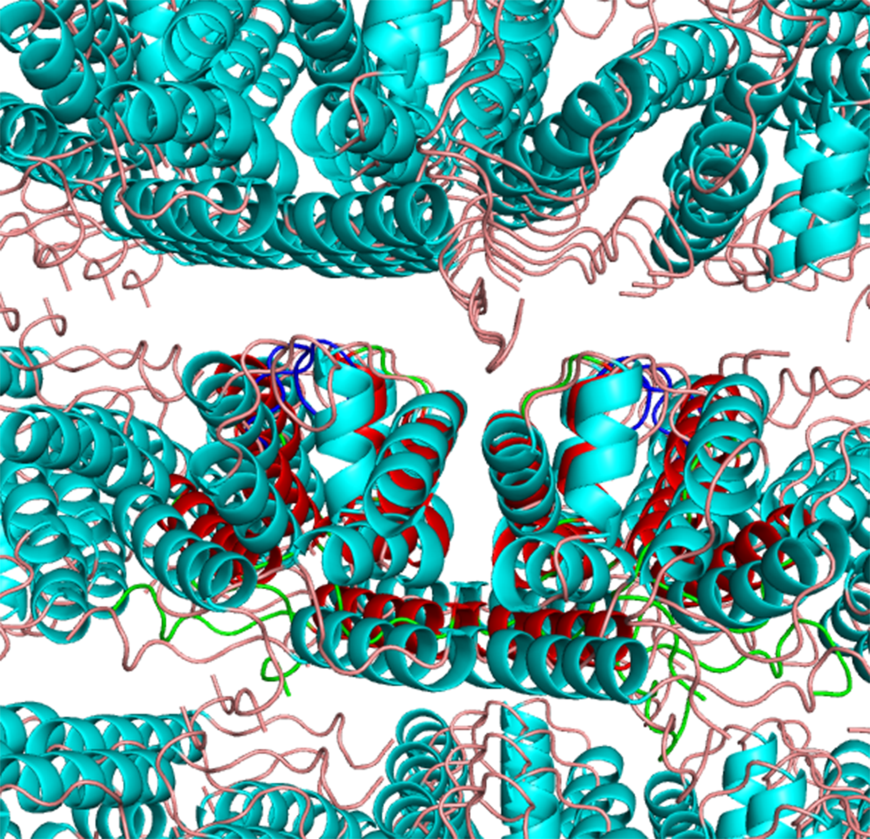
**

**Figure S1. A representative orientation showing the crystal packing for AtClpD NTD molecules.** The two molecules in the asymmetric unit are shown as red helices and green loops and the loop region between the helices α2 and α3 is shown in blue. The symmetry molecules are shown as cyan helices and pink loops. The crystal packing does not appear very tight.

**
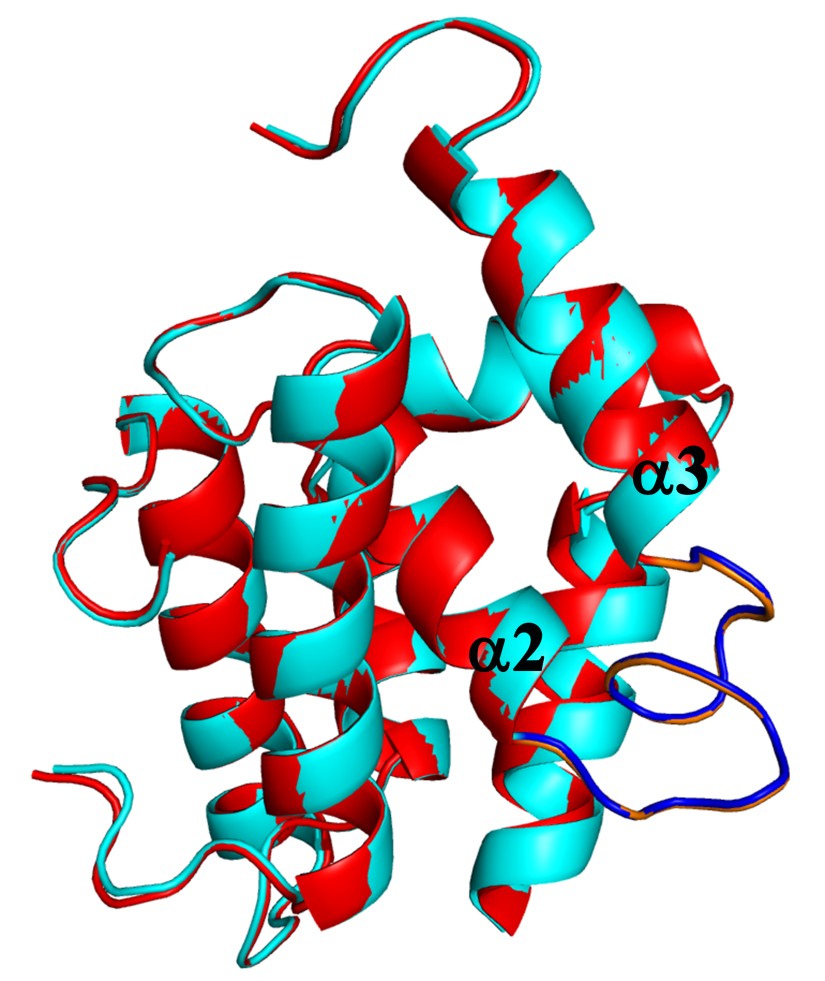
**

**Figure S2. Structural alignment of the two molecules in the asymmetric unit for AtClpD NTD crystal structure in *P*2_1_ space group**. One molecule is shown in red and the other in cyan and the loops between helices α2 and α3 of the respective molecules are shown in orange and blue respectively. For clarity purpose, only α2 and α3 are labelled. The two molecules reveal almost identical structures; aligning with an r.m.s. deviation of 0.19 Å for 112 Cα atoms. It can be seen that the loop between α2 and α3 for the two molecules align very well with each other.


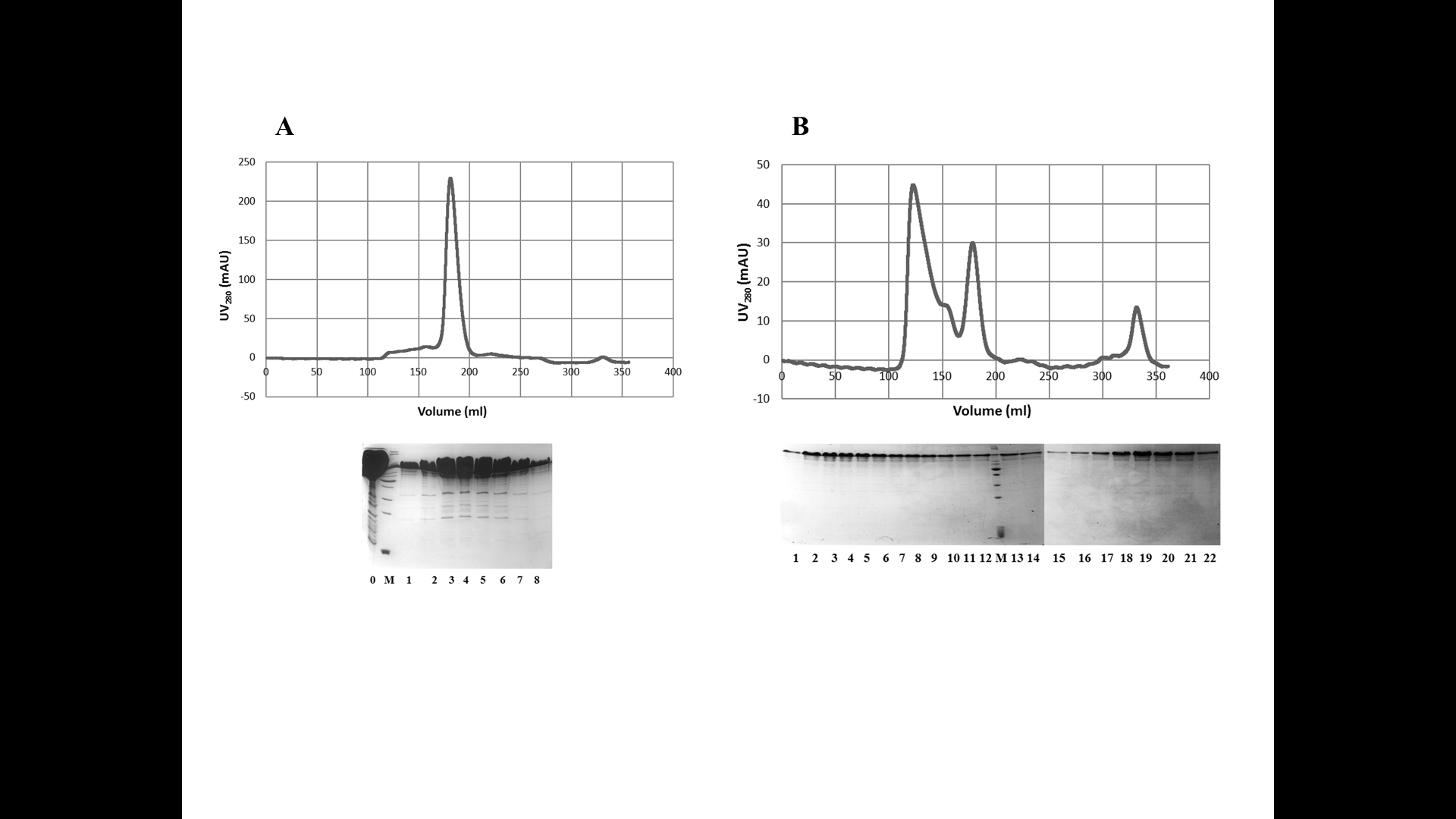


**Figure S3. Gel-filtration chromatography profiles for the purification of AtClpC1 and AtClpD full-length proteins and the corresponding SDS-PAGE images**. Gel-filtration was done using a HiLoad 26/600 Superdex 200 prep grade column. Under our expression and purification conditions, AtClpD seems to form a hexamer more readily than AtClpC1. **(A)** AtClpC1 gave a major peak corresponding to its monomeric size and the SDS-PAGE image has fractions between 165 ml to 210 ml volume during the fractionation in lanes 1 to 8. Lane-0 corresponds to the input protein after affinity purification. **(B)** AtClpD gave a major peak corresponding to its hexameric size, followed a small peak (dimer) and a peak for its monomeric size. The SDS-PAGE images have fractions between 110 ml to 190 ml volume during the fractionation in lanes 1 to 22.


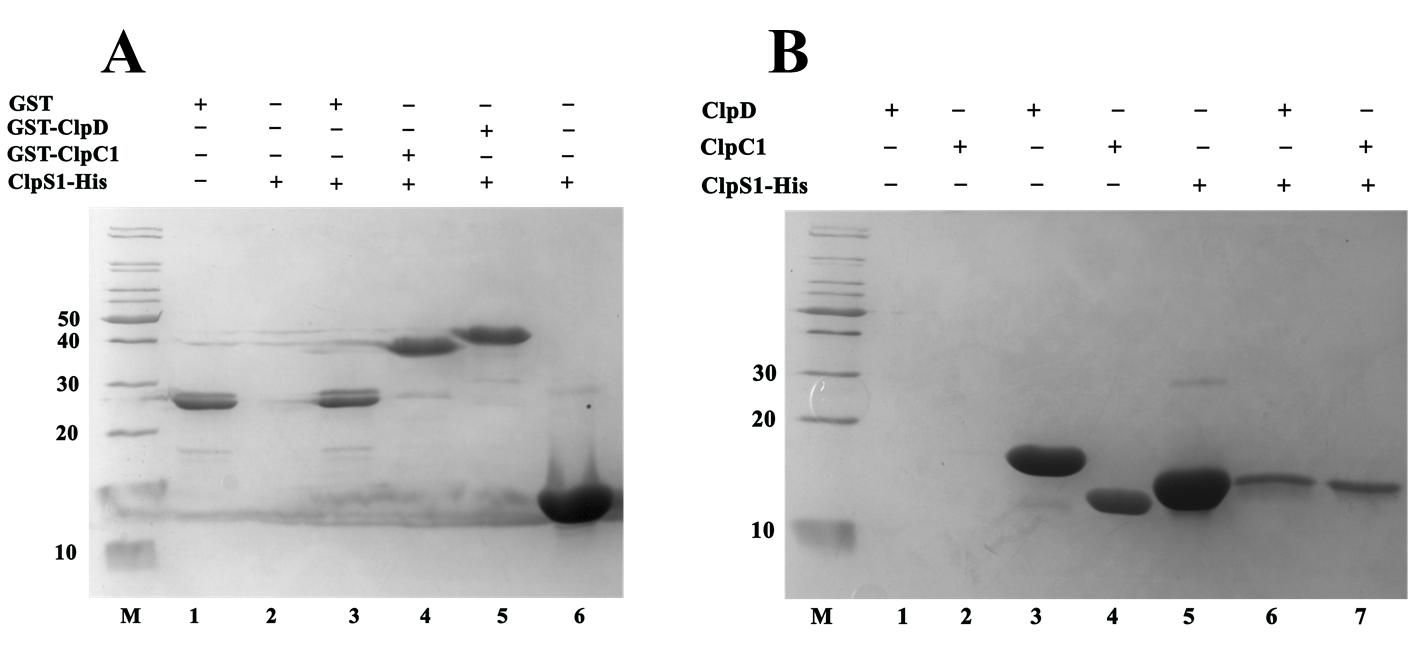


**Figure S4. SDS-PAGE images for pull-down assays**. Lane M corresponds to marker and the molecular weight of the bands are given in kDa. **(A)** Pull-down with Glutathione sepharose beads. Lane-6 is the ClpS1-His input, loaded as a control. **(B)** Pull-down with Talon resin beads. Lanes 3, 4 and 5 have the input proteins, loaded as control.


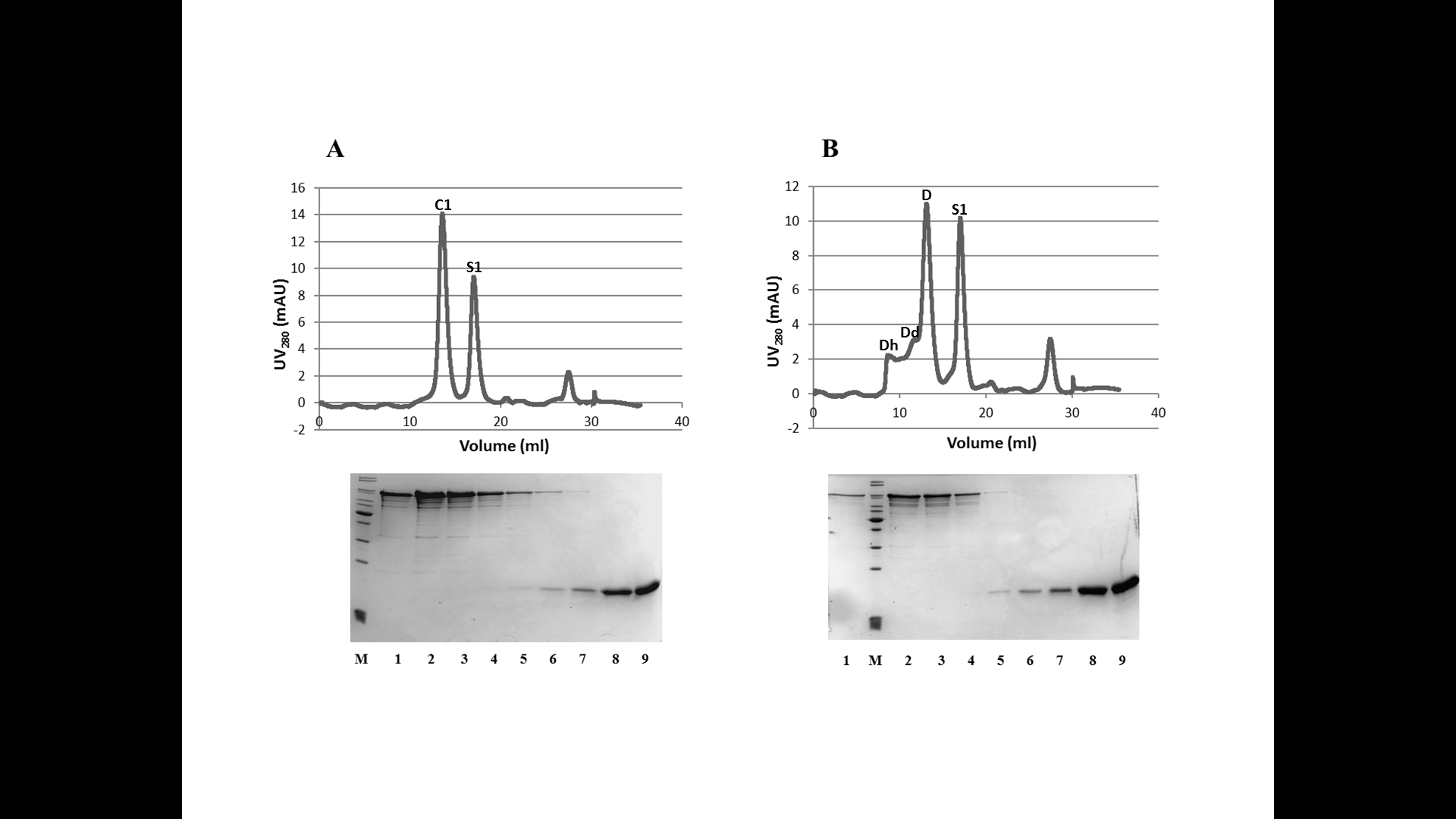


**Figure S5. Analytical gel-filtration chromatography profiles for AtClpC1 and AtClpD full-length proteins in monomeric form with AtClpS1 and the corresponding SDS-PAGE images**. Chromatography was done using a Superdex 200 10/300 GL column. **(A)** AtClpC1 with AtClpS1 gave separate peaks (labelled as C1 and S1), indicating no interaction with each other. The corresponding SDS-PAGE image has fractions between 12.5 ml to 17.5 ml volume during the fractionation in lanes 1 to 9. A little of AtClpS1 coming along with the trailing fractions of AtClpC1 is thought to be because of a certain degree of peak overlap due to slow fractionation of AtClpC1. **(B)** AtClpD with AtClpS1 also gave separate peaks for the two (labelled as D and S1), indicating no interaction. AtClpD also gave a small percentage as hexamer and dimer (labelled as Dh and Dd, respectively). The corresponding SDS-PAGE image has fractions between 12 ml to 19.5 ml volume during the fractionation in lanes 1 to 9. Even in the presence of 0.5 mM EDTA, a small percentage of AtClpD came out in oligomeric forms.


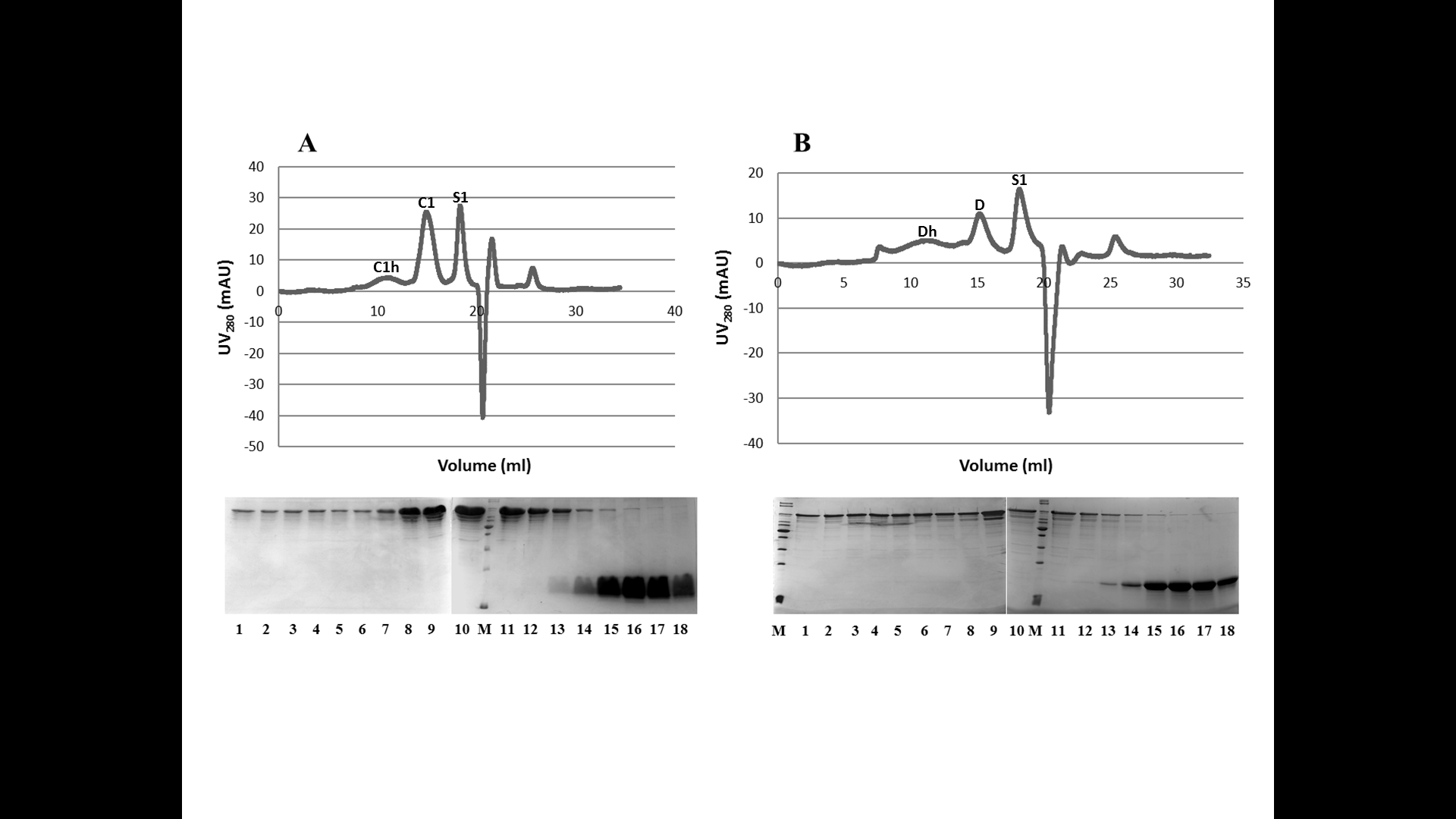


**Figure S6. Analytical gel-filtration chromatography profiles for AtClpC1 and AtClpD full-length proteins in hexamerization condition with AtClpS1 and the corresponding SDS-PAGE images**. Chromatography was done using a Superose 6 10/300 GL column. The sharp negative spike in the profiles corresponds to the component of hexamerization condition (perhaps, AMP-PNP). Hexamerization of the two proteins were not complete. A little of AtClpS1 coming along with the trailing fractions of AtClpC1/D monomer is thought to be because of a certain degree of peak overlap due to slow fractionation of AtClpC1/D. **(A)** AtClpC1 hexamer and AtClpC1 monomer (labelled as C1h and C1) gave separate peaks and these were separate from AtClpS1 (labelled as S1) also; indicating no interaction between AtClpC1 and AtClpS1. The corresponding SDS-PAGE image has fractions between 10.0 ml to 19.0 ml volume during the fractionation in lanes 1 to 18. **(B)** AtClpD gave a slightly more pronounced peak for hexameric form (labelled as Dh). However, even the monomeric fraction (labelled as D) gave a separate peak from AtClpS1 (labelled as S1), indicating no interaction. The corresponding SDS-PAGE image has fractions between 9.5 ml to 19.0 ml volume during the fractionation in lanes 1 to 18.
